# Trait emotional intelligence and ecological outcomes: the role of connectedness to nature

**DOI:** 10.1186/s40359-024-01679-9

**Published:** 2024-04-12

**Authors:** Vanessa Marchetti, Angelo Panno, Massimiliano Scopelliti, Luciano Romano, Giacomo Angelini, Elena Rinallo, Daniela Barni, Caterina Fiorilli

**Affiliations:** 1 Department of Human Studies, Lumsa University of Rome, Rome, Italy; 2 Experimental and Applied Psychology Laboratory, Department of Human Studies, European University of Rome, Rome, Italy; 3 Department of Human and Social Sciences, University of Bergamo, Bergamo, Italy

**Keywords:** Climate change, Connectedness to nature, Love and care for nature, Pro-environmental behaviors, Trait emotional intelligence

## Abstract

**Background:**

Global climate change is recognized as a major and irreversible challenge for humanity, requiring people’s responsible and sustainable behaviors toward the environment. So far, the literature has widely investigated the role of cognitive determinants of ecological outcomes (e.g., pro-environmental behaviors and climate change perception), while less attention has been devoted to emotional processes, such as trait emotional intelligence (TEI). The current double study investigates whether TEI is directly and indirectly associated with climate change perception (CCP, Study 1) and pro-environmental behaviors (PEBs, Study 2) among young adults. Furthermore, the mediating role of connectedness to nature (CN), both as cognitive and emotional factors, was also analyzed. We hypothesized that CN (i.e., cognitive mediator) would positively mediate the relationship between TEI and CCP (H1), and Love and Care for Nature (LCN, i.e., emotional mediator) would positively mediate the relationship between TEI and PEBs (H2).

**Methods:**

The study involved 342 young adults (F = 60.7%; age 19–40; M_age_=22.99; SD = 2.66) in Study 1 and 365 young adults (F = 71.2%; age 17–35; M_age_=22.2; SD = 3.98) in Study 2. Data were collected through an online tool shared by the snowball method. We administered the following self-reports: Trait Emotional Intelligence Questionnaire- Short Form (TEIQue- SF), Global Climate Change (GCC), and Connectedness to Nature Scale (CNS) (Study 1); Trait Emotional Intelligence Questionnaire- Short Form (TEIQue-SF), General Environmental Behaviors Scale (GEB), and Love and Care for Nature (LCN) (Study 2).

**Results:**

Findings from Study 1 showed that higher TEI levels enhance CN (i.e., cognitive mediator), positively influencing CCP (estimate = 0.14; 95% CI = 0.07 to 0.23). Findings from Study 2 showed that higher TEI levels are associated with higher LCN levels (i.e., emotional mediator), influencing people’s engagement in PEBs (estimate = 0.7; 95% CI = 0.03 to 0.11).

**Conclusion:**

It is crucial to design environmental education programs that promote greater emotional intelligence ability and encourage individuals’ involvement in ecological outcomes.

## Introduction

It is widely recognized that most environmental issues, including climate change, environmental pollution, and biodiversity loss, are dominantly due to human behaviors [1]. Environmental research suggests structural environmental problems may seriously harm global humanity [2, 3]. Furthermore, the United Nations [4] has mentioned environmental degradation as one of humanity’s top ten greatest threats.

These alarming data push us not to disregard the ecological issue by investigating personal characteristics more likely to be associated with ecologically responsible behaviors. In this regard, cognitive variables, such as attitudes [5], personal values referring to self-transcendence [6], altruistic values [7], family values [8, 9], and personal norms [10, 11] have been investigated, while less attention has been devoted to people’s trait emotional intelligence (TEI). The present study aims to contribute to a better understanding of individuals’ climate change perception (CCP) and their engagement in pro-environmental behaviors (PEBs) by investigating whether trait emotional intelligence (TEI) can act as a precursor to individuals’ CCP and, subsequently, their involvement in PEBs, with the mediating role of connectedness to nature (CN). Due to people’s TEI associations with several life outcomes (e.g., pro-social behaviors, academic and work-life engagement, mental and physical health [12–15]), it is expected to find associations with positive attitudes toward the environment in terms of interest, sensitivity, and responsible acts to safeguard the ecological balance between humans and the planet. Nevertheless, until now, few studies have investigated these relationships, which may add a new approach to explaining people’s differences in ecological thoughts, emotions, and behaviors. Our two studies addressed this issue by exploring the associations between people’s TEI and ecological outcomes (i.e., climate change perception and pro-environmental behaviors).

### People’s trait emotional intelligence: the unexplored link with ecological outcomes

Trait emotional intelligence (TEI; [16]) is a theoretical framework that focuses on assessing and understanding emotional intelligence as a stable personality trait. It encompasses four key dimensions: well-being, self-control, emotionality, and sociability. Well-being refers to one’s ability to recognize and regulate emotions in oneself and others, manage stress, and maintain a positive emotional state. Self-control involves managing impulsive feelings and behaviors, demonstrating restraint, and resisting immediate gratification. Emotionality refers to the extent to which individuals are comfortable experiencing and expressing emotions, and it includes understanding and interpreting emotional signals from oneself and others. Finally, sociability captures one’s interpersonal skills and the ability to navigate social situations effectively. It involves empathy, understanding others’ emotions, and building positive relationships [13, 14, 17]. The central key to TEI is people’s perceived ability to manage and regulate emotions and cope with challenging, stressful, and emotional situations. TEI can influence individuals’ attitudes, values, and decision-making processes [18], which may affect their ecological behaviors. Until now, no previous studies have investigated whether TEI is associated with people’s positive attitudes and behaviors toward nature (i.e., climate change perception and pro-environmental behaviors).

Climate change perception (CCP) refers to the extent to which an individual perceives, through various sources of information, the risks that the planet is facing [3, 19]. Different studies have focused on whether, where, and how people acquire information about CCP, their main concerns, and how judgments about the risk to the planet and human lives are formed (e.g., [19–21]). Moreover, people’s CCP is strongly associated with positive attitudes toward nature [18, 22–24], making the construct particularly relevant for further investigation due to its practical implications.

A recent literature review has shed light on how emotions influence people’s responses to climate change [25]. For example, Van Valkengoed and Steg [26] conducted a meta-analysis in which the predictive role of emotions experienced by individuals on their judgments related to climate change emerged. Furthermore, consistent with other studies, negative emotions (such as anger, fear, and contempt) strongly predict the perception of climate change risk [27, 28]. In general, the literature is consistent on the crucial role of emotions in fostering assumptions of responsibility, risk perceptions, and conscious attention toward climate change (e.g., [25, 29]). Nevertheless, feeling emotively involved differs from emotional ability as encompassed in TEI. Effectively, personality traits could be significantly informative in understanding peoples’ differences in their ecological outcomes. In this vein, it is interesting that Panno and colleagues [30] have recently shown that two personality factors (i.e., openness to experience and honesty-humility) are related to ecological outcomes through moral anger. However, to our knowledge, the links between people’s TEI (for example, peoples’ emotional management, emotional awareness, empathy, and sociability) and CCP remain less investigated.

Pro-environmental behaviors (PEBs) refer to actions taken by individuals to minimize their negative impact on the environment and promote sustainability. More specifically, PEBs are conceived regarding a bundle of specific behaviors that are different in terms of financial cost, effort, knowledge, and other factors [31]. This behaviors include both acts that benefit the natural environment (e.g., recycling) and the omission of acts that hurt it (e.g., avoiding using a car). Although there is a growing consensus that people’s PEBs are crucial in issues related to the Earth’s safeguarding, a relatively small number of people are willing to adapt their lifestyles to reduce one ecological impact significantly. Previous studies on emotions and nature can be divided into two strands: those dedicated to emotional experiences in natural contexts, which have focused on children (0–10 years), young adults (10–24 years) [32], and the adult population[35], and those focused on individual emotional factors capable of predicting PEBs (as proved in systematic literature reviews [33, 34]). Findings consistently agree about the positive associations between people’s emotional experiences, such as awe, wonder, joy, and tranquility, and their positive attitudes toward nature as well as their preferences for natural environments (e.g., [32–35]).

### The mediating role of feeling connectedness to nature

People’s TEI is strictly related to self-control, emotional management, empathy, social awareness, and optimism (e.g., [36]). With this in mind, new links not yet investigated are expected to be found, such as people’s sensitivity to the environment and care and love toward natural dimensions. According to several authors, two main constructs are used to measure how people feel in a community with nature: connectedness to nature (CN; [37]) and love and care for nature (LCN; [38]). Mayer and Frantz [37] have proposed the concept of connectedness to nature and its measurement, the Connectedness to Nature Scale (CNS), referring to the individual’s feelings of being in a community with nature, and it consists of values-based attitudes and personal beliefs, which are part of one’s self-concept. Perrin and Benassi [39] state that CNS captures a cognitive dimension of one’s involvement with the natural world. CNS is rooted in Leopold’s [40] argument that individuals must establish a profound connection with the broader natural environment to address environmental challenges effectively [37]. According to Leopold, this involves evaluating the degree to which individuals perceive themselves as equal members within the wider natural community, recognizing themselves as an integral part of the natural world, just as it is an essential part of them, and acknowledging the interdependence between their well-being and the well-being of the natural world.

Another dimension to evaluate people’s feelings toward the natural world is Love and Care for Nature (LCN; [38]). It refers to the individual’s emotional connection, appreciation, and concern for the natural world. It encompasses affection, respect, and a sense of responsibility towards nature and its preservation. This characterization encompasses the subsequent theoretical components, primarily drawn from philosophical sources: (1) enduring sentiments of reverence, astonishment, and fascination towards nature, which are described as emotions that elicit a sense of concern; (2) emotions of affection, emotional intimacy, and interconnection with nature, encompassing a spiritual facet somewhat overlooked within the field of psychology; and (3) sentiments of concern, duty, and dedication to safeguard the natural world [38]. Even though, to our knowledge, no previous studies have directly investigated the links between TEI and individual connection with nature, some research would seem to suggest this association. For example, Di Fabio and Bucci [41] have found a significant association between empathy (i.e., a component of TEI) and CNS among Italian high school students. Findings concerning the relationships between emotional intelligence or emotions and LCN are lacking. Some authors investigating such a relationship strictly focused on people’s feelings toward the natural world. For instance, in a study involving a sample of 238 adults from the UK, it was found that LCN was associated with ethically conscious consumption choices [42], and in another study that involved 454 young adults from five countries, the authors demonstrated that nature exposure promotes individuals’ well-being through feelings of connection with nature [43]. In contrast, no previous studies focused on the link between people’s TEI and LCN.

### The present study

Previous research has supported two main assumptions. First, TEI is a constellation of emotional self-perceptions located at the lower levels of personality hierarchies [16, 44]. Second, people’s TEI affects their pro-social behaviors (i.e., [45– 49]). With this in mind, the current study investigates the relationship between TEI and ecological outcomes, such as PEBs and CCP, in two young adult samples. Furthermore, according to the above literature, people’s connectedness to nature (via cognitive and emotive approaches) is expected to mediate the relationship between the studied variables. More in detail, Study 1 examines the relationship between TEI and CCP through the connectedness to nature, assumed to capture people’s cognitive involvement toward nature. Study 2 investigates the indirect relationship between TEI and PEBs via an emotional connection to nature, namely love and care for nature (LCN). We set the following hypotheses.

Study 1. H1: We predicted that TEI would be related to CCP through CN. More specifically, we expected that higher levels of TEI would be related to higher levels of CN, which, in turn, would enhance CCP.

Study 2. H2: We predicted that TEI would be related to PEBs through LCN. More specifically, we expected that higher levels of TEI would be related to higher levels of LCN, which, in turn, would enhance PEBs.

#### Study 1

The aim of the first study is to investigate the relationship between trait emotional intelligence (TEI), connectedness to nature (CN, focused on a cognitive approach), and climate change perception (CCP). The following hypothesis has been formulated:

H1: We predicted that TEI would be related to CCP through CN. More specifically, we expected that higher levels of TEI would be related to higher levels of CN, which, in turn, would enhance CCP.

## Method

### Participants

The study involved 342 Italian participants aged 19 to 40 (M_age_=22.99, SD = 2.66). In detail, 207 (60.7%) of participants identified themselves as women, and 134 (39.3%) identified themselves as men. They were workers and university students from Northern, Central, and Southern Italy. The inclusion criteria for the study were (1) being a Italian young adult (aged between 17 and 40 years) and (2) voluntarily agreeing to participate.

### Procedure and measures

Data were collected through an online questionnaire administered via the Limesurvey platform, which did not allow the respondent to proceed if the fields were not completed. For this reason, there was no missing data. The study adopted a convenience sample, and participants were recruited via networking through friends, colleagues, and casual acquaintances. Only overage participants who gave informed consent were involved in the study, and anonymity and confidentiality standards were ensured at every data collection stage. The study was conducted under the Declaration of Helsinki and approved by the Ethics Committee of LUMSA University (protocol code 4/2023 and date of approval 02/05/2023).

#### Trait emotional intelligence

Trait emotional intelligence was measured by the Trait Emotional Intelligence Questionnaire-Short Form (TEIQue-SF; [50, 51]). It is a self-report measure of 30 items assessing the four domains of TEI: Well-Being (e.g., “Overall, I am happy with my life”), Self-control (e.g., “Overall, I can cope with stress”), Emotionality (e.g., “It is not difficult for me to put my emotions into words”) and Sociability (e.g., “I can interact well with others”). Items are rated on a 7-point Likert scale (1 = completely disagree, 7 = completely agree). In the current study, Cronbach’s alpha was 0.83 for the total score; in the original study, Cronbach’s alpha was 0.89 for the total score.

#### Cognitive connectedness to nature

The Connectedness to Nature Scale (CNS; [37, 52]) evaluates people’s connectedness with nature as a cognitive dimension. It comprises 14 items rated on a 5-point Likert scale (1 = completely disagree, 5 = completely agree). An example of an item is: “I feel as though I belong to the earth as equally as it belongs to me”. In the present study, Cronbach’s alpha was 0.88; in the original study, Cronbach’s alpha was 0.82 for the total score.

#### Climate change perception

People’s climate change perception was measured by three items from Heath and Gifford’s [53] questionnaire, namely the Global Climate Change (GCC). The three items of GCC are evaluated on a 5-point Likert scale (1 = strongly disagree, 5 = strongly agree). Specifically, items were as follows: “It seems that weather patterns had changed compared to when I was a child”; “It seems to me that temperature is warmer now than in years before”; and “I have already noticed some signs of global warming”. In the present study, Cronbach’s alpha was 0.79.

### Analysis plan

Firstly, we sought to test the adequate normality of the distribution by exploring means, standard deviations, minimum and maximum, skewness, and kurtosis of the study variables. As none of the variables had skewness or kurtosis values greater than|2| [54] nor standard deviation nearly close to zero, the normality of the distribution was assumed. Consequently, a Pearson correlation was performed to test the association between the variables. Furthermore, to verify H1, a mediation model was tested using model 4 of PROCESS macro for SPSS v. 4.2 [55]. A percentile bootstrap procedure with 5000 re-sampling and a 95% confidence interval (CI) was adopted [56]. Specifically, TEIQue-SF was inserted as an independent variable, CNS as the mediator, and GCC as the dependent variable.

## Results

Table 1 reports the descriptive statistics and the correlation matrix.

**Table 1:** Descriptive statistics and correlations

	M	SD	Min-Max	SK	KU	2	3
1. TEIQue-SF	3.54	0.45	1.90–4.67	-0.24	0.18	0.247^**^	0.210^**^
2. CNS	3.30	0.72	1.50-5	0.04	-0.46		0.351^**^
3. GCC	3.98	0.85	1.33-5	-0.65	-0.10		

Note. **p <.01. SK = Skewness, KU = Kurtosis, TEIQue-SF = Trait Emotional Intelligence Questionnaire- Short Form, CNS = Connectedness to Nature Scale, GCC = Global Climate Change

Pearson’s correlation matrix results showed that TEIQue-SF is positively and significantly associated with CNS and GCC. Furthermore, findings show a significant positive association between CNS and GCC (Table 1).

**Fig. 1:**
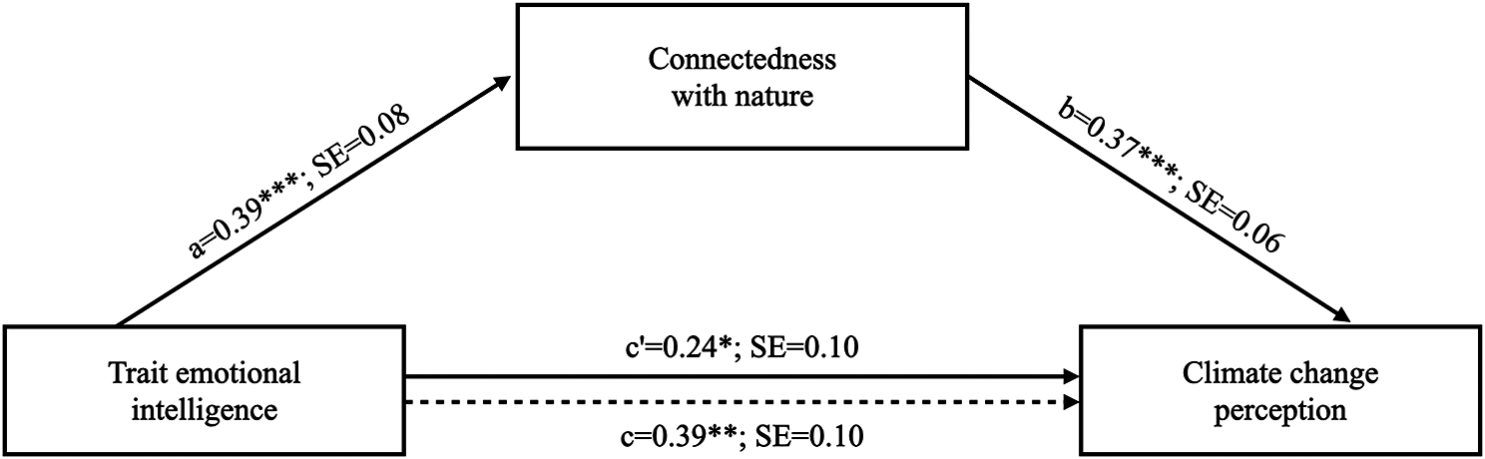
Path coefficients for the mediation model. Note. *p <.05, **p <.01, ***p <.001. The dotted line denotes the relationship between TEIQue-SF and GCC when CNS is not included as a mediator; a, b, c, and c’ are unstandardized regression coefficients

The results of the mediation model tested are depicted in Fig. 1.

We estimated the indirect effect of trait emotional intelligence on climate change perception, quantified as the product of the ordinary least square (OLS) regression coefficient by estimating CNS from TEIQue-SF (path a, in Fig. 1) and the OLS regression coefficient estimating GCC from CNS, controlling for TEIQue-SF (path b, in Fig. 1). A 95% percentile bootstrap confidence interval (CI) for the product of these paths that do not include zero provides evidence of a significant indirect effect [56]. Results showed a significant positive indirect association of TEIQue-SF with GCC through CNS (point estimate = 0.14; 95% CI = 0.07 to 0.23). Results from study 1 and 2 will be discussed in the same section.

### Study 2

The second study aims to analyze the relationship between trait emotional intelligence and pro-environmental behaviors through Love and Care for Nature. The following hypothesis was formulated:

H2: We predicted that TEI would be related to PEBs through LCN. More specifically, we expected that higher levels of TEI would be related to higher levels of LCN, which, in turn, would enhance PEBs.

## Method

### Participants

The study involved 365 Italian young adults (71.2% women, 27.7% men, 1.1% preferred not to specify) aged 17 to 35 years (M_age_=22.2; SD = 3.98). Workers and university students from Northern, Central, and Southern Italy participated. The inclusion criteria for the study were (1) being a Italian young adult (aged between 17 and 40 years) and (2) voluntarily agreeing to participate.

### Procedure and measures

Data were collected through an online questionnaire administered via the Google Forms platform, which did not allow the respondent to proceed if the fields were not completed. For this reason, there was no missing data. Participants individually completed the questionnaire on a laptop computer or a smartphone, and the anonymity of their responses was guaranteed. As for Study 1, the study adopted a convenience sample, and participants were recruited via networking through friends, colleagues, and casual acquaintances. The study was conducted following the Declaration of Helsinki and approved by the Ethics Committee of LUMSA University (protocol code 4/2023 and date of approval 02/05/2023). Concerning underage participants, only those with parental consent were allowed to participate in the study. The questionnaire was organized into different sections: the first part provided the informed consent form and general compilation instructions, and the second included the scales for measuring the relevant constructs.

#### Trait emotional intelligence

As in Study 1, we adopted the Trait Emotional Intelligence Questionnaire - Short Form to assess participants’ trait emotional intelligence (TEIQue-SF; [50, 51]). Items were rated on a 5-point Likert scale (1 = completely disagree, 5 = completely agree). In the present study, Cronbach’s alpha was 0.87; in the original study, Cronbach’s alpha was 0.89 for the total score.

#### Pro-environmental behaviors

The short version of the General Environmental Behaviors Scale Italian adaptation (GEB; [9, 57, 58]) measured people’s pro-environment behaviors. The 30-item scale provides an assessment of PEBs, grouped into four domains: Strong Ecological Behavior (e.g., “I am a member of an environmental organization”), Sustainability in Everyday Life (e.g., “After one day of use, my sweaters or trousers go into the laundry.”), Recycling and Reduced Waste Production (e.g., “If possible, I buy products in refillable packages”), Sustainable Mobility (e.g., “I usually ride a bicycle, take public transportation or walk to go to the university/at work”). Ratings were made on a 5-point Likert-type scale, ranging from 1 = “completely disagree” to 5 = “completely agree”. In the present study, Cronbach’s alpha was 0.80 for the total score; in the original study, Cronbach’s alpha was 0.80 for the total score.

#### Emotional connectedness to nature

The Love and Care for Nature scale (LCN; [38]) was administered to assess participants’ emotional feelings toward the natural world. The LCN scale consists of 15 items. Examples are: “I feel content and somehow at home when I am in unspoiled nature” and “When I am in natural environments, I feel emotionally connected to nature.” Ratings were made on a 5-point Likert-type scale, ranging from 1 = “completely disagree” to 5 = “completely agree.” In the present study, Cronbach’s alpha was.94; in the original study, Cronbach’s alpha was.97 for the total score.

### Analysis plan

Similar to Study 1, firstly, we attested the normality of the distribution by exploring means, standard deviations, minimum and maximum, skewness, and kurtosis of the study variables. Given the normality of the distributions, Pearson’s correlation was adopted to examine the associations among the study variables. Furthermore, to verify H2, a mediation model was tested using model 4 of PROCESS macro for SPSS v. 4.2 [55]. As for Study 1, a percentile bootstrap procedure with 5000 re-sampling and a 95% confidence interval (CI) was adopted [56]. In detail, we inserted TEIQue-SF as the independent variable, LCN as the mediator, and GEB as the dependent variable.

## Results

Table 2 reports the descriptive statistics and Pearson’s correlation matrix.

**Table 2:** Descriptive statistics and correlations

	M	SD	Min-Max	SK	KU	2	3
1. TEIQue-SF	3.50	0.52	1.73–4.97	-0.265	-0.01	0.20^**^	0.08
2. LCN	3.83	0.83	1–5	-0.443	-0.227		0.42^**^
3. GEB	3.31	0.49	1.53–4.67	-0.086	-0.35		

Note. **p <.01. SK = Skewness, KU = Kurtosis, TEIQue-SF = Trait Emotional Intelligence Questionnaire- Short Form, LCN = Love and Care for Nature, GEB = General Environmental Behaviors Scale

The correlation results showed that TEIQue-SF is positively and significantly associated with LCN. Furthermore, findings showed a significant positive association between LCN and GEB.

**Fig. 2:**
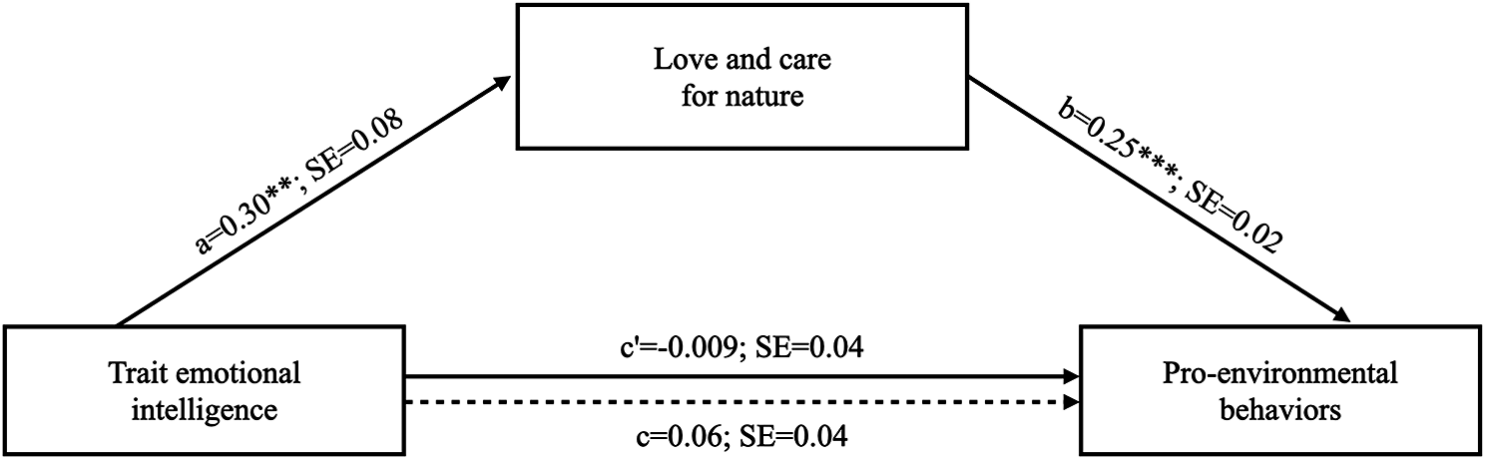
Path coefficients for the mediation model. Note. *p <.05, **p <.01, ***p <.001. The dotted line denotes the relationship between TEIQue-SF and PEB when LCN is not included as a mediator; a, b, c, and c’ are unstandardized regression coefficients

A graphical representation of the results of the mediation model tested is reported in Fig. 2.

To test the hypothesized model, we estimated the indirect effect of TEIQue-SF on GEB, quantified as the product of the ordinary least square (OLS) regression coefficient estimating LCN from TEIQue-SF (path a, in Fig. 2) and the OLS regression coefficient estimating GEB from LCN, controlling for TEIQue (path b, in Fig. 2). Results showed a significant positive indirect association between TEIQue-SF and GEB through LCN (point estimate = 0.07; 95% CI = 0.03 to 0.12)^1^.

## Discussion

This study analyzed the relationship between TEI and people’s attitudes and behavior toward nature, considering the mediation role of peoples’ feelings in community with nature. Given TEI’s comprehensive nature encompassing cognitive, emotional, and social aspects, it holds significant potential to deeply understand individuals’ overall involvement towards ecological behaviors and attitudes. Moreover, the personality trait nature of TEI, such as stable emotional traits, leads to speculation on possible antecedents of peoples’ responsible behaviors toward the environment.

Study 1’s correlation findings showed that greater levels of TEI are positively associated with the cognitive aspects of CN. Moreover, the CN is positively associated with people’s CCP. Concerning Study 2, results showed that the more people’s TEI increases, the more their LCN level grows. Furthermore, LCN is positively associated with people’s PEBs. In contrast, no significant correlations have been found between TEI and PEBs. This result is counterintuitive since previous studies conducted in different countries and among people of various age groups (e.g., among Italian high school students [13, 14], Italian adult workers, aged 27-65, and among Australian adultswith a mean age of 34,17 years [15]) have shown that individual with high TEI levels are more likely to engage in pro-social behaviors, empathy toward others, and nurte caring for their relationships. Nevertheless, the further found correlations between TEI and studied variables (i.e., CN, CCP, LCN), not previously investigated, which led us to sustain that people with high TEI levels are also expected to show high levels of involvement in sensitive and responsible interests as well as positive attitudes toward ecological issues.

Considering the investigated mediating roles, our findings confirm the expected models. First, in Study 1, CN significantly mediates the associations between people’s TEI and CCP. This novelty result, not previously investigated, confirms the antecedent role of people’s TEI. It is partially in line with Panno and colleagues’ [61] study, where a positive relationship between cognitive reappraisal (i.e., an emotion regulation strategy) and ecological outcomes (i.e., CCP and PEBs) through intensive negative emotions, has been found. Furthermore, our data confirm Di Fabio and Bucci’s study [41], showing that TEI (in that case, empathy) can activate peoples’ feelings of connectedness with nature. Furthermore, Study 1 has demonstrated that sensitivity to news and experiences related to CCP, which constitutes personal awareness and attention to the surrounding environment [62], is also the result of an individual’s sense of CN.

Second, people’s sensitivity to ecological issues is further investigated in Study 2. Our findings show that the strength of the relationship is enhanced when LCN has been introduced as a mediator. Previous studies have largely demonstrated that peoples’ emotions are positively associated with different responsible behaviors towards the environment, such has been found in a sample of 175 Spanish young adult undergraduates and recent graduates (mean age of 25.76 years) [36], in another study that involved 688 healthy adults from Spain (mean age of 36.02 years) [63], in a sample of 7704 adults from London (aged between35 to 64 years) [64] and in a meta-analytic investigation across 7898 participants (aged between 11 to 51 years) from different countries [65]. However, our study further supports the scarce findings regarding the role of people’s emotional intelligence components as possible predictors of their PEBs [66]. The mediating role of LCN adds a novelty perspective to this scenario.

Furthermore, the two studies shed light on emerging controversies regarding conceptualizing CN [39, 67]. Evidence showed that cognitive and emotional CN approaches can enhance people’s CCP (Study 1) and PEBs (Study 2).

Finally, CN plays a crucial role in this scenario. Indeed, in light of the findings from our study, CN (via emotional and cognitive approach) mediates the effect of TEI on ecological outcomes, even when TEI does not directly affect engagement in PEBs, as in the case of Study 2. Conversely to our second hypothesis (H2), TEI indirectly affects PEBs via the effect that TEI has on CN. These findings show how TEI is fundamental in developing people’s ecological involvement (such as Love and Care for Nature), which, in turn, may increase people’s PEBs.

### Limitations

The two studies are not without limitations. Firstly, self-report questionnaires might undermine the validity of the results due to social desirability bias [68, 69]. The literature has stressed that sometimes self-reports may fail to reflect objective behavior accurately [70, 71]. Furthermore, online completion of the questionnaires can be considered a limitation due to the impossibility of ensuring the process was followed by each participant. Secondly, the cross-sectional nature of the present research prevents any causal interpretation, even though these studies offer helpful insights into these relations. Third, some of our results may be limited in representativeness and generalizability because data was collected using convenience samples (non-probability). Fourth, our study did not include some socio-demographic information (such as marital status) and some socio-economic information (such as social background and political orientation) on the sample, which has been widely investigated in the literature with regard to ecological outcome (e.g., [72–76]), therefore might therefore have possible implications for the results. Finally, analyses of two different samples may prevent us from determining whether there is a direct link between the global CCP and the subsequent involvement of individuals in behaviors to protect the environment [77]. Since several authors highlight such a link, it was beyond the scope of the current research to investigate it. Rather, we preferred to focus on using different samples and measures of CN and the outcome variables to support the validity and generalizability of the findings. Nonetheless, they provide a good starting point for improving future research. Indeed, adopting two distinct samples represents a methodological decision of considerable importance that enhances the generalizability of results and presents various significant implications. First, it provides a more detailed and articulated understanding of the relationship between TEI, CN, and ecological outcomes through different contexts and among heterogeneous demographic groups. Second, these results become more representative, facilitating the formulation of conclusions that can be applied with greater reliability to a wide range of situations and populations, thus enriching the value of our findings. Third, results from two samples can contribute more effectively to developing policies, interventions, and educational strategies.

### Future directions

The novelty of our results concerns the contribution in exploring the link between TEI and ecological outcomes, which has, until now, been unexplored and, thus, represents one of the reasons for their relevance. However, these preliminary results offer the potential for different perspectives in future studies. Future research should adopt direct measures of peoples’ behaviors to mitigate social desirability bias due to self-report questionnaires. Furthermore, it would be interesting to investigate, through a longitudinal design, the nature and complexity of the relationship between the mediators and the respective dependent variables tested and whether the observed changes persist over time. Moreover, future longitudinal studies might help corroborate TEI impact as an antecedent variable. Furthermore, we encourage future research through an experimental design, which could test whether the observed effects result from a causal relationship between the variables. Finally, expanding the study to focus on high school students would also be interesting, as would replicating the research design in different international contexts.

## Conclusion

Emphasizing the role of TEI provides insights into the predictors of ecofriendly outcomes (e.g., CCP and PEBs). It sheds light on the possibility of intervening to enhance emotional skills early on. Although the TEI construct is considered relatively stable over time [22, 78], it is the result of slow and multifaceted growth in individuals who can receive educational stimuli from a young age to develop emotional control, foster sensitivity toward others and the environment, and ultimately improve emotional regulation abilities such as impulsivity or selfishness (e.g., [79]). In line with this reasoning, it can be considered that high levels of TEI, due to their significant protective effect on oneself and the environment, should be central in educational interventions from school and parenting perspectives (e.g., [66, 80, 81]). Furthermore, given the crucial role of connectedness to nature, including this construct in educational programs also becomes relevant [78]. These results, therefore, hold the potential to contribute to the design of environmental education programs that nurture improved emotional management, emotional recognition, and empathy.

## Data Availability

The datasets used and/or analyzed during the current study are available from the corresponding author upon reasonable request.

## Abbreviations

CN: connectedness to nature
CNS: Connectedness to Nature Scale
CCP: climate change perception
GCC: Global Climate Change
GEB: General Environmental Behaviors Scale
LCN: Love and Care for Nature
PEBs: pro-environmental behaviors
TEI: trait emotional intelligence
TEIQue-SF: Trait Emotional Intelligence Questionnaire-Short Form

## Footnotes

1 Hayes and other authors (e.g [59]), recommend that “Researchers not require a significant total effect before proceeding with tests of indirect effects. A failure to test for indirect effects in the absence of a total effect can lead to you miss some potentially interesting, important, or useful mechanisms by which X exerts some kind of effect on Y” ( [60] p. 414).
